# Gasdermin-D Genetic Knockout Reduces Inflammasome-Induced Disruption of the Gut-Brain Axis After Traumatic Brain Injury

**DOI:** 10.3390/ijms26083512

**Published:** 2025-04-09

**Authors:** Erika d. l. R. M. Cabrera Ranaldi, Helen M. Bramlett, Oliver Umland, Leo I. Levine, Robert W. Keane, Juan Pablo de Rivero Vaccari, W. Dalton Dietrich, Nadine A. Kerr

**Affiliations:** 1 Department of Neurological Surgery and The Miami Project to Cure Paralysis, University of Miami Miller School of Medicine, Miami, FL 33136, USA; edc74@miami.edu (E.d.l.R.M.C.R.); hbramlett@med.miami.edu (H.M.B.); lil25@miami.edu (L.I.L.); rkeane@med.miami.edu (R.W.K.); jderivero@med.miami.edu (J.P.d.R.V.); ddietrich@med.miami.edu (W.D.D.); 2 Bruce W. Carter Department of Veteran Affairs Medical Center, Miami, FL 33136, USA; 3 Diabetes Research Institute, University of Miami Miller School of Medicine, Miami, FL 33136, USA; oumland@med.miami.edu; 4 Department of Cellular Physiology and Molecular Biophysics, University of Miami Miller School of Medicine, Miami, FL 33136, USA

**Keywords:** inflammasome, pyroptosis, interleukin, IL-1β, traumatic brain injury, exosomes, extracellular vesicles, gut dysfunction

## Abstract

Traumatic brain injury (TBI) pathology is significantly mediated by an inflammatory response involving inflammasome activation, resulting in the release of interleukin (IL)-1β and pyroptotic cell death through gasdermin-D (GSDMD) cleavage. Inflammasome components are transported through extracellular vesicles (EVs) to mediate systemic inflammation in peripheral organs, including the gut. The purpose of this study was to determine the protective effect of GSDMD knockout (KO) on TBI-induced inflammasome activation, EV signaling, and gut function. GSDMD-KO and C57BL6 (WT) mice were subjected to the controlled cortical impact model of TBI. Cytokine expression was assessed with electrochemiluminescent immunoassay and immunoblotting of the cerebral cortex and gut. EVs were examined for pathology-associated markers using flow cytometry, and gut permeability was determined. GSDMD-KO attenuated IL-1β and IL-6 expression in the cerebral cortex and reduced IL-1β and IL-18 in the gut 3 days post-injury. GSDMD-KO mice had decreased neuronal- and gut-derived EVs compared to WT mice post-TBI. GSDMD-KO EVs also had decreased IL-1β and different surface marker expression post-TBI. GSDMD-KO mice had decreased gut permeability after TBI. These data demonstrate that GSDMD ablation improves post-TBI inflammation and gut pathology, suggesting that GSDMD may serve as a potential therapeutic target for the improvement of TBI-associated pathologies.

## 1. Introduction

Traumatic Brain Injury (TBI) patients suffer from systemic complications, which contribute to morbidity and mortality [1]. Approximately 89% of severe TBI patients show signs of non-neurological organ dysfunction, which are associated with worse outcomes [2]. TBI survivors are more likely to suffer from digestive complications compared to healthy individuals [3]. One of the commonly overlooked systemic complications after TBI is disruption of the bidirectional brain–gut axis. Approximately 50% of severe TBI patients experience feeding intolerance, which is correlated with injury severity [3]. Gastrointestinal (GI)-related issues that arise in severe TBI patients include gut dysbiosis, gastroparesis, and impaired lower esophageal function [4]. Homeostasis of the GI system is important in the regulation of intestinal barrier function, gut motility, and neurobehavior which can all of which can be disrupted after TBI [5]. Although the clinical consequences of brain injury on gut function are well described, limited research has been conducted to critically evaluate cellular and molecular mechanisms underlying brain–gut dysfunction after TBI.

In recent years, studies have determined that the bidirectionality of the brain–gut axis plays an important role in the progression of gut dysfunction and neurological damage after central nervous system (CNS) injury [6]. There are various pathways that contribute to gut–brain axis dysfunction after CNS injury. These pathways include the systemic immune response as well as the autonomic and enteric nervous systems, the neuroendocrine system, and the microbiome. In this regard, the gut–brain axis has been shown to play an important role in innate immunity [7]. The role of the inflammasome, a component of the innate immune system, has been implicated in the gut–brain axis in CNS disorders such as multiple sclerosis, Parkinson’s disease, and Alzheimer’s disease [8]. The inflammasome is a multiprotein complex, which activates the pro-inflammatory cytokines interleukin (IL)-1β and IL-18. Our previous studies have demonstrated that inflammasome activation contributes to TBI-induced systemic organ damage in organs such as the lungs [9] and the heart [10]. Activation of the inflammasome leads to a caspase-1 mediated form of cell death known as pyroptosis. Gasdermin-D (GSDMD) is a pore-forming protein that upon activation, which forms membrane pores through N-terminus oligomerization, leading to pyroptosis and the release of inflammatory cytokines, including IL-1β and IL-18 [11,12]. Recently, we reported that pyroptosis plays an important role in the bidirectionality of gut–brain axis disruption after ischemic stroke [13]. Regarding systemic injury, others have demonstrated that genetic ablation of GSDMD reduces lung inflammation, cell death, and pathology associated with hyperoxic lung injury [14]. In addition, we have established that extracellular vesicles (EVs) are released after CNS injury [15] and isolated from circulating fluids contain inflammasome proteins that contribute to systemic organ dysfunction after brain injury [9,13].

EVs are lipid-bound vesicles ranging in size from 10 to 1000 nm that are secreted from almost all cell types and found in bodily fluids, transferring proteins, lipids, and genetic materials between cells [16]. Following TBI, evidence shows that the circulating EV population increases and changes after injury [17]. We have previously reported that EVs isolated from severe TBI patients with acute lung injury have increased neuronally derived and lung-derived EVs [18]. We have also shown that when EVs derived from the serum of mice who have undergone ischemic stroke and are injected into naïve mice, there is increased pyroptosis in the gut [13]. Analysis of circulating EV-content after TBI has also shown that IL-1β is increased post-injury [19]. Furthermore, EVs derived from gut microbes are capable of crossing the blood–brain barrier (BBB) and the intestinal epithelial barrier [20]. However, the role of TBI-induced serum-derived EVs containing inflammasome proteins as a signaling mechanism for gut–brain axis dysfunction has not been studied.

In the current study, we examined the role of plasma-derived EV-mediated pyroptosis in gut–brain axis dysfunction after TBI. We found that whole body ablation of GSDMD reduces inflammasome protein expression in cortical and intestinal tissues after TBI and that brain-derived and gut-derived EVs contain less inflammasome protein content. Finally, we report a decrease in immune cell-derived EVs in GSDMD-KO mice and an improvement in gut permeability compared to WT mice. These data support a causative role of inflammasome-induce pyroptotic cell death in the pathophysiology TBI and identify a therapeutic target for reducing the consequences of brain injury on the gut–brain axis.

## 2. Results

### 2.1. GSDMD-KO Attenuates Pro-Inflammatory Cytokine Concentration in the Cerebral Cortex 3 Days Post-TBI

**Figure 1:**
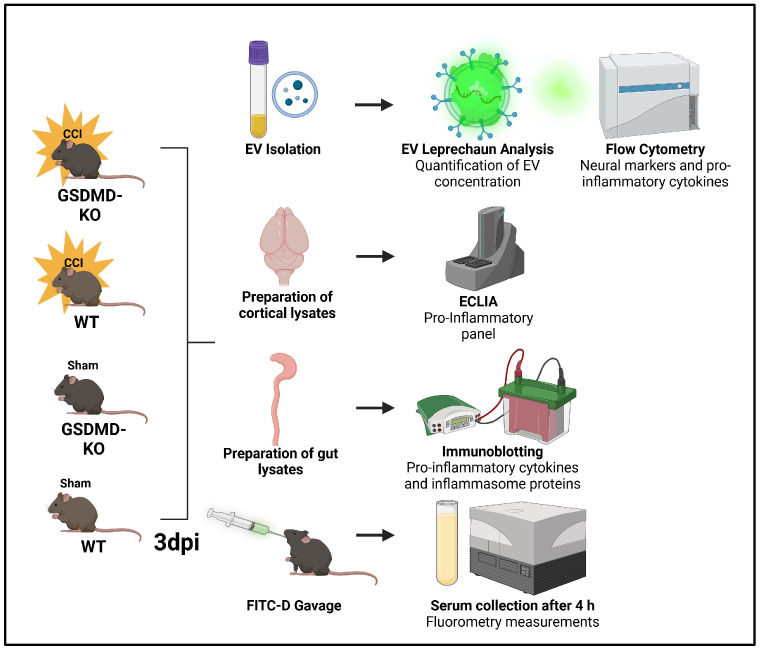
Experimental Timeline and Procedures. Five-month-old GSDMD-KO and WT animals underwent moderate controlled cortical impact (CCI) or sham surgery. The mice were sacrificed 3 days post-surgical procedures, where the ipsilateral cerebral cortex, gut, and blood were collected. The cerebral cortex and gut were homogenized and lysed, and the cerebral cortical lysates underwent probing for pro-inflammatory cytokines with ECLIA, while the gut lysates were probed for pro-inflammatory cytokines via immunoblotting. Blood plasma was used for EV isolation for flow cytometry analysis. A separate mice cohort underwent FITC-D oral gavage 3 days post-TBI, then were sacrificed 4 h post-administration and the serum collected. Serum fluorescence intensity changes were used as a proxy for gut permeability changes using spectrofluorometry.

**Figure 2:**
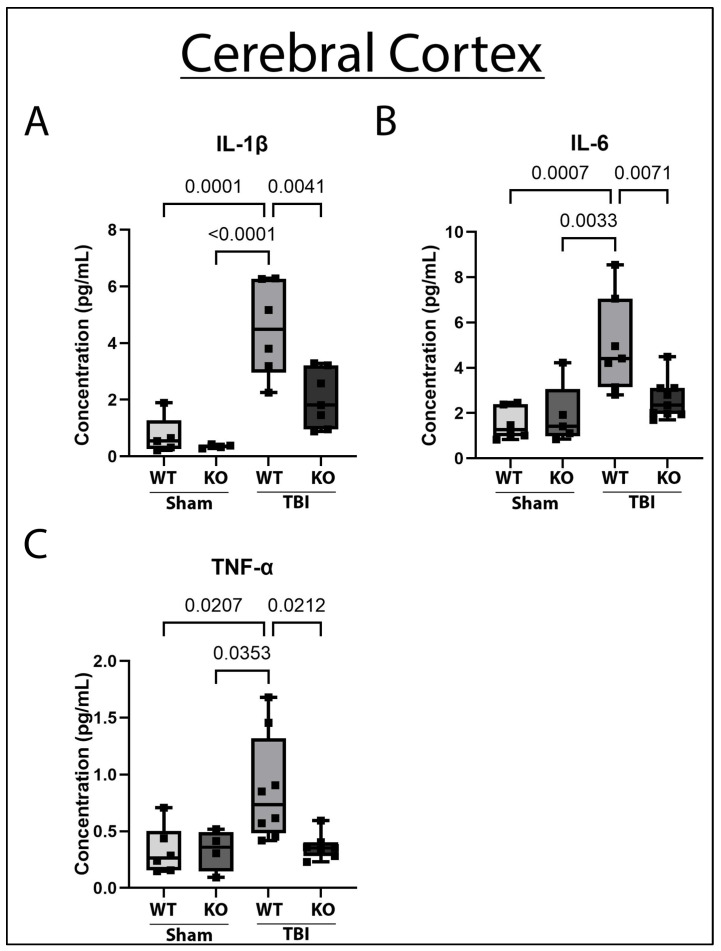
GSDMD-KO Improves TBI-Induced Elevation in Pro-Inflammatory Cytokine Expression in the Cerebral Cortex 3-Days Post-TBI. (**A**) WT TBI was present with increased levels of expression of the pro-inflammatory cytokine IL-1β 3 days post-injury. Mice with GSDMD-KO with moderate TBI had a significant reduction in IL-1β to sham levels 3 days post-TBI in the cerebral cortex. (**B**) GSDMD-KO mice who underwent TBI had attenuated protein expression of the pro-inflammatory cytokine IL-6 in the cerebral cortex in comparison to WT mice who underwent TBI 3 days post-injury. (**C**) GSDMD-KO mice had decreased concentration of TNF-α in the cerebral cortex 3 days post-TBI in comparison to WT mice. The data were normalized to total protein concentration. One-Way ANOVA and the Kruskal–Wallis test were used for normally distributed or not normally distributed data, respectively. Tukey’s post hoc multiple comparisons test was used. Significant *p*-value was set to *p* < 0.05. N = 4–9 per group.

Previous work has demonstrated that pro-inflammatory cytokines, such as IL-1β and IL-6, are increased in the cerebral cortex acutely after TBI [21,22]. Increased pro-inflammatory cytokines contribute to cerebral edema, cortical volume loss, and cognitive impairment post-TBI [23]. Because GSDMD is an important modulator of pro-inflammatory cytokine release, we first determined whether GSDMD-KO affected pro-inflammatory cytokine levels in the cerebral cortex after TBI. To this end, 5-month-old GSDMD-KO and WT animals underwent sham or moderate TBI (Figure 1) and 3 days post-TBI, ipsilateral cerebral cortices were dissected, homogenized, and lysed. Cortical lysates were probed for IL-1β, tumor necrosis factor-α (TNF-α), and IL-6 protein concentration levels via ECLIA. Statistical analyses demonstrated that moderate TBI increased acute IL-1β, TNF-α, and IL-6 cytokine concentration in the cerebral cortex of WT mice compared to both sham groups. Interestingly, in GSDMD-KO mice, the cortical concentrations of IL-1β, IL-6, and TNF-α post-TBI were significantly reduced when compared to sham-operated mice (Figure 2). Therefore, GSDMD-KO successfully protected against the rise in the pro-inflammatory cytokines IL-1β, TNF-α, and IL-6 in the cerebral cortex acutely after TBI.

### 2.2. IL-18 and IL-1β Are Decreased in the Gut of GSDMD-KO Mice 3-Days Post-TBI

**Figure 3:**
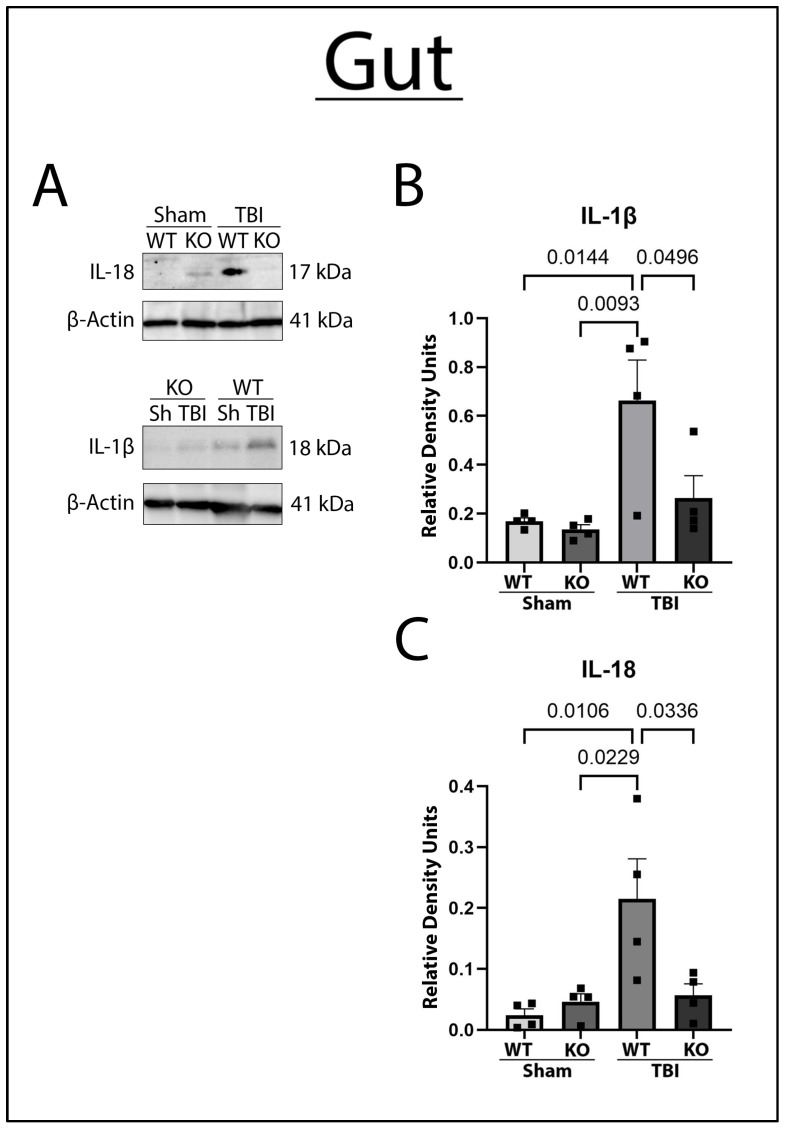
TBI Results in Greater Pro-Inflammatory Cytokine Expression in the Gut 3-Days Post-TBI, which is Attenuated by GSDMD-KO. (**A**) Representative images of immunoblots of gut lysates in post-TBI WT and GSDMD-KO mice normalized to β-actin. (**B**) WT TBI had higher expression of IL-1β in the gut 3 days post-TBI; GSDMD-KO mice had a decreased expression of IL-1β back to sham levels. (**C**) There was a significant elevation of the expression of the pro-inflammatory cytokine IL-18 in the gut of WT TBI mice, and GSDMD-KO exhibited a marked reduction in IL-18 levels in comparison. One-Way ANOVA and the Kruskal–Wallis test were used for normally distributed or not normally distributed data, respectively. Tukey’s post hoc multiple comparisons test was used. Significant *p*-value was set to *p* < 0.05. N = 4 per group.

To examine the effect of GSDMD-KO on gut inflammasome protein and pro-inflammatory cytokine levels after TBI, gut lysates of KO and WT mice who underwent sham or moderate CCI were immunoblotted for IL-18, and IL-1β. There was a significant decrease in gut IL-18 and IL-1β pro-inflammatory cytokine levels in the GSDMD-KO mice compared to WT mice 3 days post-TBI (Figure 3), suggesting that GSDMD activity is important for gut inflammatory activity post-TBI. Furthermore, the ablation of the GSDMD mechanism can protect against pro-inflammatory cytokine expression in the gut post-TBI.

### 2.3. Brain-Derived and Gut-Derived EVs Are Reduced in the Plasma of GSDMD-KO Mice After TBI

**Figure 4:**
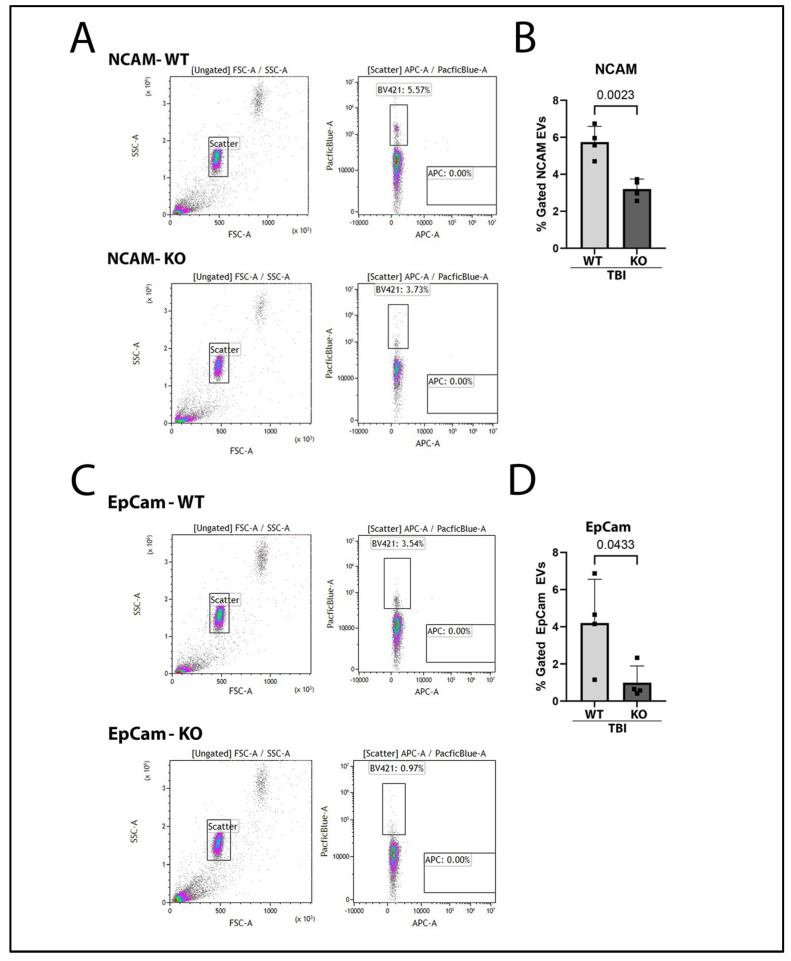
GSDMD KO Leads to Decreased Brain-Derived and Gut-Derived EVs in the Blood Plasma 3-Days Post-TBI. (**A**) Isolated EVs from the plasma of WT TBI and GSDMD-KO TBI were captured with the MACSplex EV IO kit capture beads and then stained for NCAM (**A**,**B**) and EpCam (**C**,**D**) expression for flow cytometry analysis. (**A**) Representative images of flow cytometry gates are shown with capture beads on the left plots (PE/FITC), and the right plot representing capture beads gated for those expressing NCAM (BV421). (**B**) Flow cytometry events were quantified through comparison of the percent gated NCAM EVs between GSDMD-KO TBI and WT TBI. GSDMD-KO-derived EVs had reduced NCAM expression versus EVs derived from WT mice isolated 3 days post-TBI. (**C**) Representative images of flow cytometry gates are shown with capture beads on the left plots (PE/FITC), and the right plot representing capture beads gated for those expressing EpCam (BV421). (**D**) Percent gated EpCam EVs were lowered with GSDMD-KO 3 days post-TBI. The data were analyzed via a two-way Student’s *t*-test, with significant *p*-value set to *p* < 0.05. N = 4 per group.

EVs are released into the peripheral circulation after TBI [17]. EVs are important for cell communication and are capable of enveloping inflammatory molecules, such as cytokines, and of transporting them as cargo to various organ systems such as the lungs and gut [24,25,26]. EVs can be released from the CNS and can cross the BBB and circulate within the blood [27]. Neural cell adhesion molecule (NCAM) is a neuronal-derived protein that can act as a marker for EVs originating from the CNS [28]. Previous research has demonstrated that NCAM-positive EVs are released after TBI into plasma [18]. Thus, to investigate whether GSDMD-KO affected NCAM-labeled EV release, plasma was collected from KO and WT mice 3 days after TBI. EVs were isolated from plasma using magnetic beads, stained against NCAM, and assessed via flow cytometry. Notably, NCAM-positive EVs were significantly reduced in the plasma of GSDMD-KO mice after TBI in comparison to WT mice (Figure 4B).

In addition to assessing brain-derived EV release after TBI, we also determined whether gut-derived EV release was affected by GSDMD KO. EVs were isolated from plasma of WT and KO mice after TBI, then stained against the gut-epithelial marker epithelial cell adhesion molecule (EpCam) [29]. Gut-derived EpCam-labeled EVs were then quantified by flow cytometry. There was a significant reduction in EpCam-positive EVs in KO mice compared to WT after TBI (Figure 4D). Therefore, our results indicate that GSDMD-KO reduces gut-derived EV release into the systemic circulation after TBI.

### 2.4. IL-1β Containing EVs Are Mitigated in GSDMD KO Mice 3 Days After TBI

**Figure 5:**
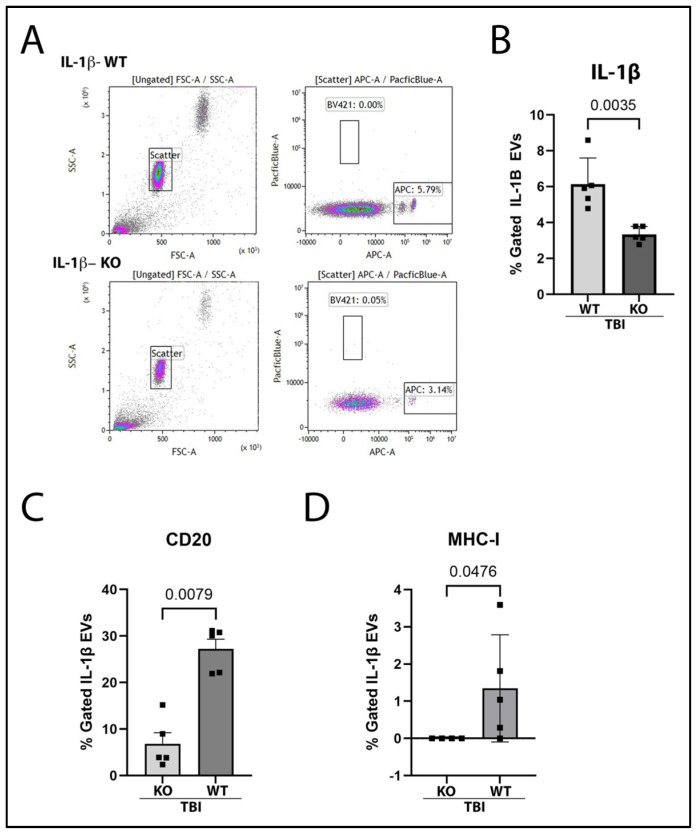
IL-1β-Containing EVs Are Diminished in the Blood Plasma of GSDMD-KO Mice 3-Days Post-TBI and Have Reduced Expression of CD20 and MHC-I 3-Days After TBI. EVs isolated from the plasma of WT TBI and GSDMD-KO TBI mice were captured with the MACSplex EV IO kit capture beads and then stained for IL-1β expression for flow cytometry analysis. (**A**) Representative images of flow cytometry gates are shown with capture beads on the left plots (PE/FITC), and the right plot representing capture beads gated for those expressing IL-1β (APC). (**B**) GSDMD-KO mice who underwent TBI have lessened percent gated IL-1β gated EVs versus WT mice 3 days post-TBI. (**C**) IL-1β-containing EVs from WT mice had greater CD20 expression than EV from GSDMD-KO mice 3 days post-TBI. (**D**) IL-1β-containing EVs from GSDMD-KO mice had decreased expression of MHC-I in comparison to EV isolated from WT mice 3 days post-TBI. The data were analyzed via a two-way Student’s *t*-test, with significant *p*-value set to *p* < 0.05. N = 5 per group.

To understand whether GSDMD-KO would reduce the release of EVs carrying pro-inflammatory cytokines, EVs were isolated from the plasma of KO and WT mice 3 days post-TBI. Isolated EVs were probed for IL-1β by flow cytometry. Notably, there was a significant reduction in IL-1β-positive EVs in KO mice after TBI when compared to WT (Figure 5B). Moreover, there were differing surface epitope expressions between GSDMD-KO-derived and WT-derived IL-1β-containing EVs. Specifically, WT-derived IL-1β-containing EVs had significantly greater expression of CD20, a B cell specific marker, in comparison to GSDMD-KO EVs (Figure 5C). Additionally, WT-derived EVs also presented increased expression of the surface marker major histocompatibility complex-I (MHC-I) in comparison to GSDMD-KO-derived EVs post-TBI (Figure 5D).

### 2.5. GSDMD-KO Protects Against Gut Permeability Dysfunction 3 Days After TBI

**Figure 6:**
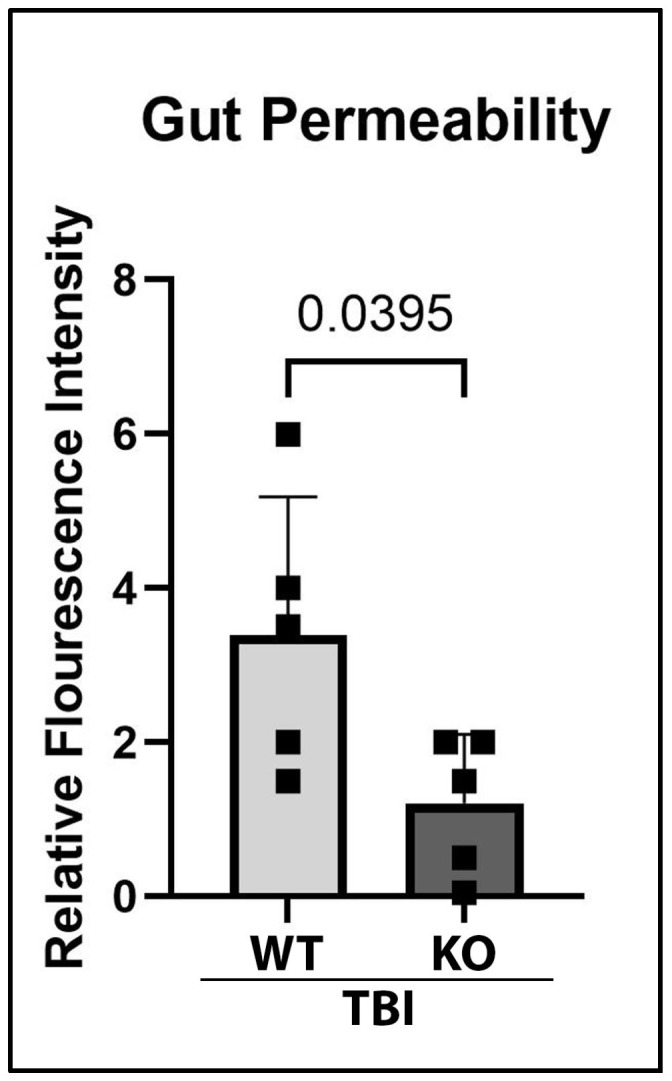
GSDMD KO Improves Impairments in Gut Permeability 3-Days Post-TBI. Gut permeability was assessed 3 days post-TBI through a FITC-D gavage assay. Fluorescence intensity in peripheral blood plasma was used to denote gut permeability. There was a marked reduction in gut permeability as quantified as fluorescent intensity in the GSDMD-KO mice in comparison to the WT mice 3 days post-injury. The data were analyzed via a two-way Student’s *t*-test, with significant *p*-value set to *p* < 0.05. N = 5 per group.

TBI can lead to gut dysfunction, with one consequence associated with TBI being increased intestinal permeability [30,31]. Permeability changes can lead to the infiltration of pathological molecules and exacerbation of inflammatory activity in the gut [32]. To assess functional recovery in the GI system after TBI, gut permeability of KO and WT mice was determined 3 days post-injury. Prior to sacrifice, animals were orally gavaged with FITC-D. After sacrifice, blood plasma was collected, and fluorescence intensity was measured as an indicator for gut permeability. Fluorescence analysis revealed that there was a significant improvement in gut permeability function after TBI in GSDMD-KO mice (Figure 6), suggesting that GSDMD inhibition can improve gut dysfunction after TBI by reducing gut permeability.

## 3. Discussion

Previous studies have demonstrated that the inflammasome pathway is one of the key players in the innate immune response after TBI [9,21,33]. The inflammasome-mediated cell death process of pyroptosis leads to the death of neurons as well as other cells, and GSDMD has been identified as the executioner of pyroptosis [34,35]. In the current study, we examined the role of plasma-derived EV-mediated pyroptosis in gut–brain axis dysfunction after TBI. Using the CCI model of TBI in WT and GSDMD-KO mice, we found that whole body ablation of GSDMD reduced inflammasome protein expression in cortical and intestinal tissues 3 days after TBI. Moreover, brain-derived and gut-derived EVs were reduced in GSDMD-KO mice compared to WT and they contained lower levels of inflammasome proteins. In addition, there was a significant decrease in immune cell-derived EVs in GSDMD-KO mice and an improvement in gut permeability compared to WT mice.

The inflammasome orchestrates the early inflammatory processes after injury via IL-1β activation through the processing of pro-caspase-1, which also results in pyroptosis [36]. Inhibition of inflammasome signaling may improve post-injury pro-inflammatory processes and recovery. For example, mice treated with an NLRP3 inflammasome inhibitor demonstrate reduced lesion volume and a reduction in pro-inflammatory cytokine expression pattern post-TBI [37]. GSDMD, a pore-forming protein that is critical for the execution of pyroptosis [21,38] is cleaved by caspase-1, leading to the breakage of the link between the N-terminus and C-terminus [39]. Caspase-mediated cleavage frees the N-terminus domain from the autoinhibition held by the C-terminus, allowing for N-terminus oligomerization and insertion into the membrane to form pyroptotic pores.

In ischemic stroke, GSDMD ablation results in infarct size reduction, inflammation, and improved survival [40]. In hyperoxic brain injury, hippocampal cell death and inflammation are attenuated by GSDMD-KO [41]. Similarly, inhibition of GSDMD activity in murine subarachnoid hemorrhage attenuates neuronal and microglial injury, abrogating pro-inflammatory activity post-injury [42]. Recently, one study showed that ablation of GSDMD attenuates motor dysfunction after TBI as well as neuropathological damage and behavioral neurological deficits [43]. Moreover, it has been reported that TBI induces activation of inflammasomes leading to increased production of inflammatory cytokines [21]. Importantly, IL-1β activity is one of the major regulators of pro-inflammatory activity post-TBI [44], leading to the release of additional pro-inflammatory molecules and aggravated pathological outcomes such as brain edema and BBB breakdown [45]. Cerebrospinal fluid (CSF) IL-1β levels have been reported to be predictive of long-term outcome post-TBI, with higher levels associated with increased mortality and worsened outcome in patient studies [46,47]. Similarly, TNF-α and IL-6 are other pro-inflammatory cytokines that are increased in the serum of TBI patients and are predictive of worse cognitive outcomes [48], as well as secondary injury processes that occur such as oxidative stress and inflammation [49]. Therefore, to determine whether GSDMD inhibition is a potential therapeutic strategy for TBI-induced inflammation, we assessed inflammatory outcomes using a murine model of moderate TBI using GSDMD-KO and appropriate WT mice. The cohort of WT mice had significantly elevated levels of the pro-inflammatory cytokines IL-1β, TNF-α, and IL-6 at 72 h post-TBI in comparison to sham operated control animals, indicating an exacerbated inflammatory response in the cerebral cortex. Notably, TBI GSDMD-KO animals did not exhibit a similar significant increase in inflammatory cytokine concentrations. In fact, pro-inflammatory cytokine levels were similar to non-injured mice controls. Furthermore, our previous work has demonstrated that TBI increases inflammatory cytokine expression up to 1-week post-injury, including IL-1β concentration in cortical tissue [33]. Thus, the ablation of GSDMD activity reduced the release of IL-1β and its downstream cytokine IL-6 post-TBI, as well as TNF-α, ameliorating the inflammatory response associated with injury.

One of the common systemic complications of TBI is disruption to the bidirectional brain–gut axis. Currently, there are no published data on the clinical use of anti-inflammasome agents on TBI patients experiencing secondary organ complications, highlighting the importance of identifying novel pathomechanisms that may contribute to TBI-induced secondary consequences including organ dysfunction. TBI survivors are more likely to suffer from digestive complications compared to healthy age-matched controls [3], and approximately 50% of TBI patients experience feeding intolerance, which is correlated with injury severity [3]. Other GI-related issues that arise in severe TBI patients include gut dysbiosis, gastroparesis, and impaired lower esophageal function [4]. Interestingly, worsened microbiome changes that occur due to gut dysbiosis have been reported to amplify brain damage after TBI [50]. Homeostasis of the GI system is important in the regulation of intestinal barrier function, gut motility and neurobehavior, all of which can be disrupted after TBI [5].

TBI increases gut permeability [51], the translocation of peripheral immune cells [52], and the release of reactive oxidative species that can enhance cell death [53] and perpetuate neuropathology [30]. We have previously reported that focal cerebral ischemia, a feature of TBI, leads to morphological damage to the gut epithelium, particularly to microvilli, 3 days after focal cerebral ischemia [13]. In this TBI study, we demonstrate, for the first time, that IL-1β and IL-18 are decreased in the gut of GSDMD-KO mice compared to WT acutely after TBI and that gut permeability, an indicator of gut function, is increased in WT mice compared to GSDMD-KO post-injury. These findings indicate that pyroptosis via GSDMD cleavage plays an important role in not only the neuroinflammatory response, but also in the damage to distal organs such as the gut, after TBI.

Previously published studies have reported an increase in inflammasome proteins in serum-derived EVs from TBI patients [54]. EVs are membrane-contained vesicles released by cells involved in the transfer of cargo from one cell to another [55]. EVs are secreted by cells into bodily fluids, including blood, stool, CSF, urine, and respiratory secretions [56,57], and play a role in pro-inflammatory and anti-inflammatory conditions, depending on their cargo [58]. After TBI, EVs released into the serum and CSF contain proteins associated with cell death and neurodegeneration post-injury in clinical studies [59]. Pre-clinically, TBI induces heightened EV release after injury, and inflammatory-mediating EV can enhance TBI neuropathology, such as lesion volume and microglial activation [17]. The current study presents novel data, demonstrating that ablation of GSDMD reduces the number of brain-derived circulating EVs. These findings also support the notion that pyroptosis, a form of programmed cell death, significantly contributes to brain and systemic damage after injury.

A crucial part of the TBI-induced systemic inflammatory response involves release of EVs that contain a cargo of inflammasome proteins that affects the brain and peripheral organs, causing tissue destruction after TBI [15,60]. Studies have demonstrated that communication between the brain and peripheral organs after injury is mediated by EV signaling [13,61,62]. After TBI, there is an increase in EVs released from the CNS, which can enter the peripheral circulation and spread inflammation throughout various organ systems by their cargo, which can include pro-inflammatory cytokines and inflammasome components [9,63]. Although the brain is immunologically privileged due to the BBB, TBI and gut dysbiosis affect BBB permeability, allowing pro-inflammatory proteins to enter and exit the CNS, resulting in exacerbated systemic inflammation after TBI. In turn, damage to the intestinal epithelial barrier allows the entry of inflammatory mediators from the brain into the gut, leading to aggravated gut inflammation. Currently, the mechanisms by which TBI leads to gut dysbiosis and how gut dysbiosis affects TBI outcomes remain poorly understood. It is known that blockage of EV-signaling activity improves outcomes in peripheral organ injury post-TBI, including acute lung injury and attenuates inflammasome activity in the brain and lung [9]. Thus, to determine whether GSDMD-KO causes changes in EV-dependent systemic inflammation, EVs isolated from KO and WT mice post-TBI were also evaluated for gut epithelial-derived EVs and inflammatory-content. In GSDMD-KO mice, the concentration of gut-epithelial derived EVs and IL-1β in circulating plasma-derived EVs was reduced compared to WT mice. This finding indicates that GSDMD ablation is capable of diminishing pathology-associated EV release into the systemic circulation and potentially involved in bidirectional gut–brain axis disruption after TBI.

Analysis of IL-1β-positive EV also revealed differences in expression of varying surface receptors between GSDMD-KO and WT animals after TBI. IL-1β-containing EVs from WT mice had significantly greater CD20 surface marker expression after TBI. CD20 is a cell specific marker [64] expressed on the surface of B cells. B-cell signaling has been demonstrated to increase after CNS injury and to contribute to pathology and pro-inflammatory signaling in mice [65,66] and in humans [67]. When B cells are depleted in CNS injury, there are improvements in neurological function, white matter injury, and cell death [65,68,69,70]. Our results elucidate a pro-inflammatory role after TBI for B-cell signaling mediated via EVs and demonstrate that GSDMD-KO can attenuate this inflammatory signaling by reducing plasma IL-1β-containing EVs. Furthermore, IL-1β-containing EV from GSDMD-KO mice were also markedly reduced in surface markers for MHC-I, an important modulator for T-cell activation [71]. MHC-I molecules can present peptides that serve to activate CD8^+^ cells, encouraging cell death and inflammation [72]. CD8^+^ cells have been detected in the ipsilateral cortex within 8 and 32 weeks after TBI [73]. Additionally, MHC-I molecules are upregulated after TBI, and MHC-I signaling pathways are similarly increased up to one month after repetitive mild TBI in mice [74]. After depleting β2-microglobulin, which is required for MHC-I surface expression, improvements in neurological and motor outcomes have been reported after TBI [73]. The current study demonstrates that in GSDMD KO mice, there is a reduction in IL-1β-containing EV from MHC-I surface receptor expressing cells, which may participate in reducing the detrimental pro-inflammatory responses following TBI.

Systemic organ dysfunction after CNS trauma involves multiple mechanisms and pathways. For example, changes to the autonomic nervous system and the innate immune response can lead to secondary GI events, including dysmotility and increased mucosal permeability [6]. Thus, in addition to emphasizing inflammasome signaling pathways, other mechanistic components that affect multiple pathways consecutively should also be addressed. Although the present study focused in GSDMD, this is not the sole Gasdermin protein that is associated with cerebral injury pathways. Mpox virus infection of astrocytes which can lead to neurological complications such as encephalitis, causes pyroptotic cell death via Gasdermin B cleavage [75]. In other conditions, Gasdermin E cleavage has been shown to be partly responsible for pyroptotic cell death in human immunodeficiency virus-associated neurocognitive disorder [76] and in amyotrophic lateral sclerosis [77]. Additionally, various Gasdermin proteins are also associated with alternative cell death pathways, such as apoptosis in cases of ischemic stroke and CNS injury, that will also be important to examine [78]. Further, while helpful for identifying mechanisms of cell injury and potential drug targets, genetic knockout models are not adaptable for translational applications. Thus, future work should focus on how pharmacological applications can be constructed from genetic models for potential clinical use.

Although we utilize both male and female animals in this investigation, there was not sufficient power to detect sex differences in GSDMD mediated pro-inflammatory protein expression signaling after TBI. We therefore combined data taken from both sexes to conduct various analyses. Previous investigations have reported that male mice have a more robust acute increase in microglia activation after TBI in comparison to female mice. Interestingly, pro-inflammatory cytokine expression in female mice show an increase in IL-1 β and TNF-a mRNA expression at acute time points in comparison to males. Future studies on TBI are required to determine whether GSDMD-KO is an appropriate therapeutic target for both sexes.

**Figure 7:**
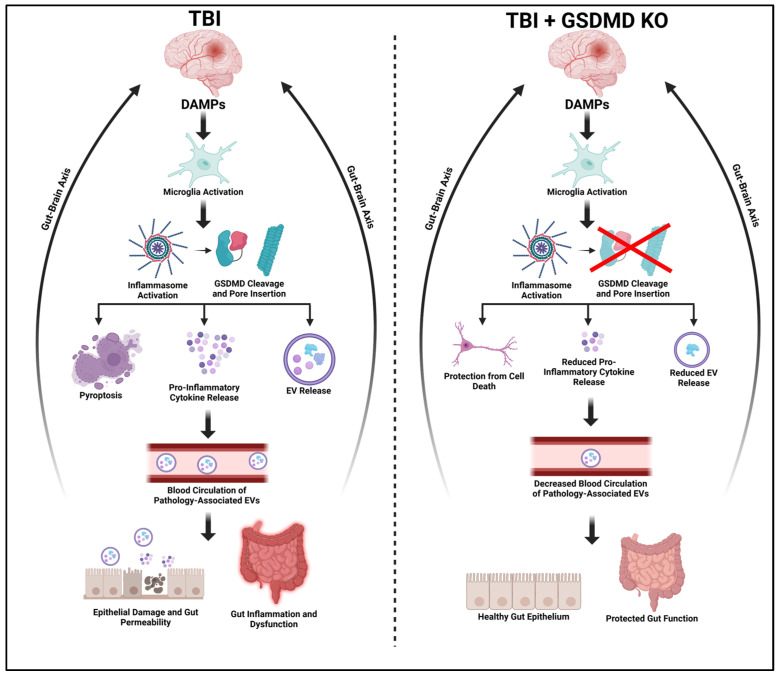
GSDMD-KO Protects Against TBI-Induced Inflammatory Signaling and Gut Pathology. Side-by-side comparison showing TBI-induced damage-associated molecular patterns (DAMPs) inducing microglial activation and inflammasome activity, which leads to GSDMD cleavage. GSDMD signaling then incites pyroptosis, pro-inflammatory cytokine release, and EV signaling. Pathology-associated EVs are released into the blood, perpetuating systemic inflammatory signaling, gut inflammation, and gut permeability. GSDMD-KO, by ablating GSDMD function, in turn protects against cell death, pro-inflammatory cytokine release, and pathological EV communication. GSDMD-KO thereby attenuates gut pathology and inflammatory processes.

In summary, we report that GSDMD genetic ablation attenuates the post-TBI inflammatory response through the reduction in proinflammatory cytokine expression in the cerebral cortex and the gut (Figure 7). Furthermore, targeting pyroptosis via a GSDMD KO approach also led to reductions in pro-inflammatory EV signaling and pathological outcomes in the gut, an organ that can be functionally affected by clinical TBI. The current finding establishes that GSDMD is an important mediator of post-TBI inflammation, and that inhibition can lead to improved outcomes in central and peripheral inflammatory activity. Therefore, therapeutic innovations targeting inflammasome-induced pyroptosis represent a promising approach for investigating and treating TBI-associated multi-organ inflammatory and pathological outcomes in the clinical setting.

## 4. Materials and Methods

### 4.1. Animal Model and Surgical Procedures

The animal procedures were completed as approved by the Institutional Animal Care and Use Committee of the University of Miami Miller School of Medicine (Animal Welfare Assurance A3224-01, #IPROTO202300002268, effective date 7 November 2023). A full-body GSDMD KO mouse model [C57BL/6N-*Gsdmd^em4Fcw^*/J] was used to assess the effect of GSDMD ablation on TBI inflammasome activation and EV signaling. GSDMD-KO mice and their respective WT controls (C57BL/6J) were organized into a 1:1 mixed sex-cohort. The number of animals per experimental group was determined by power analysis and historical data from previous experiments. All mice were 5 months of age at the time of injury with a weight range of 25 to 35 g. The mice were prospectively randomized into experimental groups. For TBI modeling, GSDMD-KO and WT mice underwent either moderate CCI or sham surgery, as described previously [33]. Briefly, the mice were anesthetized with intraperitoneal injection of ketamine and xylazine. After the proper anesthetic depth was reached, an incision was made upon the scalp, and a 5 mm craniotomy was completed over the right parietal cortex. Moderate CCI was ascertained on the exposed brain tissue at the craniotomy site with an impact of 4 m/s velocity and 0.55 mm depth over 150 ms. The scalp was stapled after impact, and the mice were administered slow-release buprenorphine subcutaneously for pain management. Sham mice received all surgical procedures with the exception to the moderate CCI. The mice were allowed to recover on a heating pad for 24 h before being returned to the animal facility. The mice were sacrificed 72 h post-surgical procedures. To increase scientific rigor, the outcome measures were carried out by an investigator blinded to the experimental groups.

### 4.2. Electrochemiluminescence Immunoassay (ECLIA)

To determine the impact of GSDMD-KO on post-TBI pro-inflammatory cytokine expression changes, ipsilateral cerebral cortices were dissected 72 h after surgical procedures after sacrifice. Cortical tissue was lysed and homogenized as detailed previously [33]. Pro-inflammatory cytokine expression was probed using the V-PLEX Proinflammatory Panel 1 Mouse Kit (Meso Scale Diagnostics, Rockville, MD, USA), as described in [79]. Briefly, prior to sample incubation, the 96-well V-PLEX plate was washed with 150 µL per well of MSD wash buffer. Then, 50 µL of cortical lysate samples and provided calibrators were added to the plate and incubated at room temperature overnight while at 700 rpm. The samples were then removed, and the plate was washed once more with an additional 150 µL of MSD wash buffer, after which 25 µL of detection antibody was added to each well for a 2 h incubation at 700 rpm. Following incubation, 150 µL of Read Buffer T (Meso Scale Diagnostics) was added and the MESO QuickPlex SQ 120 (Meso Scale Diagnostics) platform was used to quantify pro-inflammatory cytokine concentration. Protein concentration was normalized to total protein concentration as determined by a Bradford assay. Briefly, 5 µL of undiluted samples were added to a 96-well plate alongside a bovine serum albumin calibration curve, after which 145 µL of Bradford protein assay reagent (Thermo Fisher Scientific, Waltham, MA, USA #1856209) was added, and the plate was shaken to properly mix reagent and sample. The samples were then incubated for 10 min and absorbance was measured at 592 nm on the Spark microplate reader (Tecan, Männedorf, Switzerland). Calculated concentration (pg/mL) of each sample was analyzed via the Discovery Workbench software ver. 4.0 (Meso Scale Diagnostics).

### 4.3. Immunoblotting

To examine whether GSDMD-KO affected inflammasome activation after TBI, gut lysates were tested for inflammasome protein and pro-inflammatory cytokine expression. Seventy-two hours after the surgical procedures, the animals were sacrificed, and gut tissue was dissected, lysed, and homogenized as described previously [33]. Gut lysates were diluted in 4X laemmli buffer and then boiled at 100 °C for 5 min. The diluted lysates were resolved as described in [80], in 4–20% Stain Free Gel (Bio Rad, Hercules, CA, USA) for 25 min at 400 mA and 3 V. The gel was then transferred into a polyvinylidene difluoride (PVDF) membrane with the Turbo Transfer (BioRad) instrument. The membranes were immunoblotted with antibodies (1:1000 dilution in blocking buffer) against the following inflammasome-associated pro-inflammatory cytokines: IL-18 (Cell Signaling, Danvers, MA, USA #57058S) and IL-1β (Cell Signaling #12242S). The images were captured using the ChemiDoc Imaging System (BioRad) and densiometric analyses were completed using the Image Lab ver. 6.1 (BioRad) software. The total protein density was normalized to β-actin (Invitrogen, Waltham, MA, USA #MA5-15739).

### 4.4. EV Isolation

Whole blood was collected immediately after sacrifice using ethylenediaminetetraacetic acid (EDTA) syringes (Sarstedt). Plasma was isolated by centrifuging whole blood samples at 1900× *g* for 10 min at room temperature. The plasma was purified through centrifugation for 20 min at 2000× *g* at room temperature, after which the supernatant was centrifuged once more for 20 min at 10,000× *g*. EVs were isolated as described in [81] with the Total Exosome Isolation (from plasma) Kit (Invitrogen). Briefly, 50 µL of 1X PBS was added to 100 µL of plasma. Next, the samples were incubated at room temperature for 10 min with 30 µL of the Exosome Precipitation Reagent. After incubation, the samples were centrifuged at 10,000× *g* for 5 min. The supernatant was aspirated, and the exosome-containing pellet was re-suspended in 50 µL of 1X PBS.

### 4.5. EV Flow Cytometry

The isolated EVs were processed for flow cytometry analysis through the MACSPlex Exosome Kit (Miltenyi Biotec, Gaithersburg, MD, USA). The isolated EVs were diluted to 120 µL using the MACSPlex Buffer (Miltenyi Biotec). Diluted EVs were incubated overnight with 15 µL of MACSPlex EV Capture Beads at 12 rpm. After incubation, 500 µL of MACSPlex Buffer was added to the samples, which were then centrifuged for 5 min at 3000× *g.* Aspiration of 500 µL of the supernatant was completed. The samples were incubated with 10 µL of the respective antibodies: EpCam (BD Biosciences, Franklin Lakes, NJ, USA #563214), NCAM (BD Biosciences #748094), or IL-1β (Leinco, St. Louis, MO, USA #I-964) for 1 h at 450 rpm. Centrifugation was repeated, and an additional 500 µL of MACSPlex buffer was added afterwards for a 15 min incubation. The final centrifugation was completed and 500 µL of supernatant was aspirated. The remaining 150 µL of labeled EVs was transferred to a 96 clear flat-bottom plate. The samples were run on a CytoFLEX S (Beckman Coulter, Indianapolis, IN, USA). The data were analyzed through the Kaluza Analysis Software ver. 2.1 (Beckman Coulter).

### 4.6. Gut Permeability Assay

The assessment of intestinal permeability was conducted as previously described [13]. The previous literature has reported clinical gut microbiome perturbations 72 h after admission for trauma injury, including TBI; thus, 72 h was selected as the time point to explore changes in gut permeability after TBI [82]. Seventy-two hours after TBI, the mice were administered fluorescein isothiocyanate-dextran (FITC-D) 4000 (Sigma-Aldrich, St. Louis, MO, USA) via oral gavage at 60 mg/100 g of body weight. The animals were sacrificed 4 h post-gavage, and the serum was collected. The isolated serum was pipetted into a 96-well opaque plate in triplicates, along with serial dilutions of FITC-dextran in PBS to create a standard curve. A Spark microplate reader (Tecan) was used to determine sample fluorescence as an indicator of gut permeability.

## Data Availability

Available data will be provided upon contacting the corresponding author.
